# Circadian behavior of mice deficient in PER1/PML or PER2/PML

**DOI:** 10.1186/1740-3391-11-9

**Published:** 2013-08-28

**Authors:** Takao Miki, Misty Chen-Goodspeed, Zhaoyang Zhao, Cheng Chi Lee

**Affiliations:** 1 Department of Biochemistry and Molecular Biology, Medical School, University of Texas Health Science Center-Houston, Houston, TX, 77030, USA; 2 Currently at Department of Molecular Oncology, Kyoto University Graduate School of Medicine, Kyoto, 606-8501, Japan

**Keywords:** Circadian rhythm, Mouse model, Actogram, Phase shift, Period

## Abstract

**Background:**

Our recent studies demonstrate that the murine homolog of the human tumor suppressor promyelocytic leukemia (PML) regulates circadian behavior of mice. To further gather insight into PML’s contribution to circadian behavior, we generated two strains of mice deficient in one of the two period (*Per*) genes and the PML gene, with *Per1*^*−/−*^*/Pml*^*−/−*^ and *Per2*^*−/−*^*/Pml*^*−/−*^ genotypes.

**Results:**

Here we report the circadian behavior of these mice based on wheel-running behavioral analysis. In a free-running environment, the *Per1*^*−/−*^*/Pml*^*−/−*^ mice maintained circadian rhythm but displayed a significantly shorter period of 22.2 h. In addition, these mice displayed significantly enhanced phase response to a light pulse given at zeitgeber time (ZT) 14 and 22. The *Per2*^*−/−*^*/Pml*^*−/−*^ mice lose persistent rhythm when in a free-running environment, as also the case for *Per2*^−/−^ mice*.* A transient post-light pulse rhythm seen in the arrhythmic *Per2*^*−/−*^ mice was less apparent in *Per2*^*−/−*^*/Pml*^*−/−*^ mice. Both the *Per1*^*−/−*^*/Pml*^*−/−*^ and *Per2*^*−/−*^*/Pml*^*−/−*^ mice displayed a more advanced phase angle of entrainment activity during light–dark cycles than the single gene deficient mice.

**Conclusions:**

Beyond merely regulating PER1 and PER2, the current behavioral studies suggest PML has additional roles in mouse circadian behavior.

## Background

The protein, Promyelocytic leukemia (PML) has been implicated in many important biological processes, including the DNA damage response, cell division control and chromosome instability [1,2]. A reciprocal chromosomal translocation t(15;17), which fuses the *PML* and the retinoic acid receptor alpha (*RARα*) genes is the underlying cause of over 95% of acute promyelocytic leukemia (APL) cases [1]. Recently, we have shown that PML is a circadian clock regulator [3]. Loss of PML disrupts PER2 nuclear localization and dampens PER2 interaction with the clock transcriptional complex BMAL1/CLOCK, resulting in reduced heterodimer mediated transcription. Our studies show that *Pml*^*−/−*^ mice have abnormal phase shift responses to a light pulse and have circadian periods that display reduced precision and stability. The period length instability had features that were somewhat similar to the behavioral phenotype described for *Per1*^*−/−*^ mice [4,5]. At a molecular level, we observed that the loss of PML caused significantly dampened expression of *Per1*[3]. These observations raised the possibility that the phenotype of PML deficiency may be linked to deregulation of PER1 function. However, PML regulation of PER2 function could also explain the dampened *Per1* expression profile in *Pml*^*−/−*^ mice since the loss of PER2 was shown to dampen *Per1* expression [6]. To examine these possibilities, we generated *Per1*^*−/−*^*/Pml*^*−/−*^ and *Per2*^*−/−*^*/Pml*^*−/−*^ mice to investigate whether the circadian behavior of these double knockout mice was similar to that of *Pml*^*−/−*^, *Per1*^*−/−*^, *Per2*^*−/−*^ or wild type mice. Our circadian behavioral analysis based on wheel-running activities revealed that loss of PML further compromised the clock function of both *Per1*^*−/−*^ or *Per2*^*−/−*^ mice indicating that PML has a significant role in the mammalian clock mechanism.

## Materials and methods

### Animal wheel-running activity

All mice were housed in a standard animal maintenance facility under a 12 h light: 12 h dark (LD) cycle. The *Pml*^*−/−*^ (129sv) animals were obtained from the Mouse Models of Human Cancers Consortium repository, National Cancer Institute [7]. The *mPer2*^*−/−*^ (129sv/C57/b6) and *Per1*^*−/−*^ (129sv/C57/b6) mice were generated as previously described [4]. Mice deficient in either PML/PER1 or PML/PER2 were generated by initially breeding *Pml*^*−/−*^ with *Per1*^*−/−*^ or *Per2*^*−/−*^ animals to produce the heterozygote F1. The double mutant animals for *Per1*^*−/−*^*/Pml*^*−/−*^ or *Per2*^*−/−*^*/Pml*^*−/−*^ were then obtained from heterozygote F1 breeding and were genotyped by PCR with primers specific to the respective genes. The *Per1*^*−/−*^, *Pml*^*−/−*^ and *Per2*^*−/−*^ mice used for these behavioral studies were derived from this breeding scheme. Both male and female mice deficient in either *Per1*^*−/−*^*/Pml*^*−/−*^ or *Per2*^*−/−*^*/Pml*^*−/−*^ were viable, fertile and were morphologically indistinguishable from wild type. Male siblings of the respective genotypes aged about 16 weeks from the backcross were used in the wheel-running studies. Wheel-running activity was monitored as described [6]. Briefly, mice were initially entrained to a LD cycle for at least 2 weeks, followed by their release into a constant darkness free-running condition. To assess the stability of the clock, the same mice were re-entrained to an LD cycle for 2 weeks and then released again into a free-running environment. For phase shift studies, the protocol was as previously described [8,9]. Briefly, a light pulse of 15 min with an intensity of 480lux was given using white fluorescent tube light. A linear regression using data from at least 10 days following the light pulse was used to determine the level of phase shift. Zeitgeber time zero (ZT0) is light on and ZT12 is light off.

All animal experiments in this study were carried out under an institutional approved animal protocol: HSC-AWC-06-077.

## Results

### Wheel-running circadian behavior of *Per1*^*−/−*^*/Pml*^*−/−*^ mice

**Figure 1:**
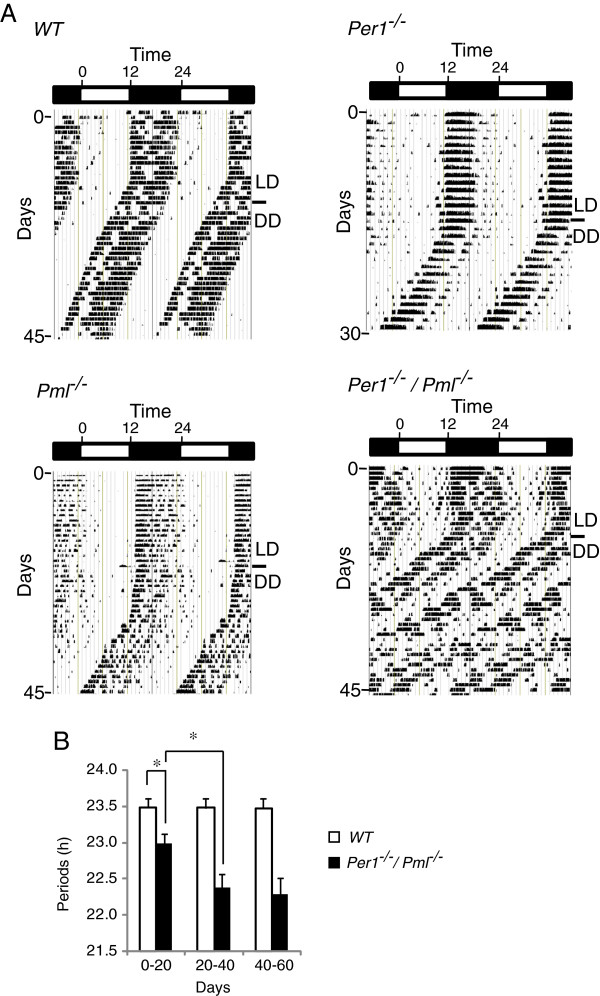
**Wheel-running behavior of *Per1***^***−/−***^***/Pml***^***−/− ***^**mice.** The wheel-running actograms are double plotted showing a 12 h light/12 h dark cycle (LD) and a free-running period of 12 h dark/12 h dark (DD) cycles. Transition from LD to DD is indicated by the marker. Actograms are representative of **(A)** a wild type mouse, a *Per1*^*−/−*^, a *Pml*^*−/−*^ and a *Per1*^*−/−*^**/***Pml*^*−/−*^ mouse. **(B)** Summary of periods obtained from wild type (n = 7) and *Per1*^*−/−*^**/***Pml*^*−/−*^ (n = 12) mice over 1–20, 20–40 and 40–60 days in a free-running environment. Error bar indicates SEM.* p < 0.05.

**Figure 2:**
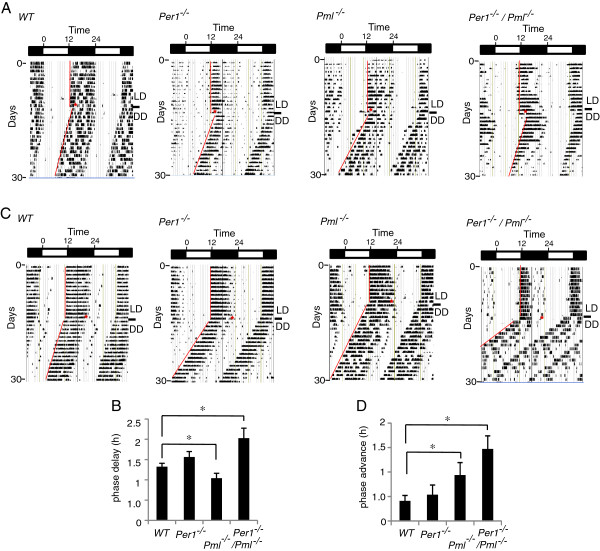
**Phase shift behavior of *Per1***^***−/−***^***/Pml***^***−/−***^**mice.** Mice were entrained to a 12 h light/12 h dark cycle (LD) and a light pulse was given on the last day of LD at ZT 14 or ZT 22. **(A)** &**(B)** Phase delay response to a ZT14 light pulse (15 min) of wild type, *Per1*^*−/−*^*, Pml*^*−/−*^ and *Per1*^−/−^/*Pml*^*−/−*^ mice. **(C)** &**(D)** Phase advance response to a ZT22 light pulse (15 min) of wild type, *Per1*^*−/−*^*, Pml*^*−/−*^ and *Per1*^−/−^/*Pml*^*−/−*^ mice. Error bar indicates SEM. p < 0.05, (n = 9).

**Figure 3:**
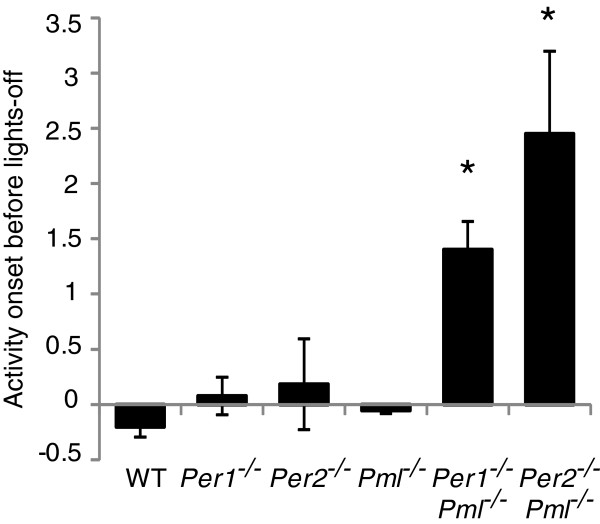
**Average time of activity onset during LD cycles.** The average time of activity onset over 14 days before ZT12 was measured for wildtype, *Per1*^*−/−*^, *Pml*^*−/−*^, *Per1*^*−/−*^*/Pml*^*−/−*^ and *Per2*^*−/−*^*/Pml*^*−/−*^ mice. Activity onset for *Per2*^*−/−*^ and *Per1*^*−/−*^ mice was previously reported by Spoelstra et al., 2004. Error bar indicates SEM. *p < 0.05, (n = 6).

Male wild type*, Pml*^*−/−*^, *Per1*^*−/−*^ and *Per1*^*−/−*^*/Pml*^*−/−*^ mice were analyzed for their circadian behavior by monitoring their wheel-running activity in a LD cycle followed by a free-running period of 12 h dark/12 h dark (DD) cycles. For all four genotypes, the mice entrained to an LD cycle and were rhythmic in the free-running environment throughout our analysis. Interestingly, when released into a free-running state, the *Per1*^*−/−*^*/Pml*^*−/−*^ mice displayed an “after effect of entrainment” where their daily period gradually shortened over the initial 20 days (Figure 1A, B). The period lengths of *Per1*^*−/−*^*/Pml*^*−/−*^ mice were eventually shortened to an average 22.2 h in the free-running environment (Figure 1B). The *Per1*^*−/−*^*/Pml*^*−/−*^ mice period lengths from day 20–40 and 20–60 were not significantly different. Under a free-running environment, the wild type, *Per1*^*−/−*^ and *Pml*^*−/−*^ mice displayed periods that averaged 23.4, 22.8 and 23.2 h, respectively (Table 1). The *Per1*^*−/−*^*/Pml*^*−/−*^ mice displayed a significant level of activity prior to light off at ZT12 during the LD cycle (Figure 1A, also see Figure 2). On average, the *Per1*^*−/−*^*/Pml*^*−/−*^ mouse phase angle of entrainment activity onset in the LD cycle started about 1.5 h prior to ZT12 (Figure 3). To a much lesser extent, the *Per1*^*−/−*^ mice displayed some activity prior to ZT12 consistent with previous observations [9]. Wild type and *Pml*^*−/−*^ mice showed no significant wheel-running activity before light off at ZT12. The current observations indicate that *Per1*^*−/−*^*/Pml*^*−/−*^ mice have a significantly advanced phase angle of entrainment compared to *Pml*^*−/−*^*, Per1*^*−/−*^ or wildtype mice during LD cycles.

**Table 1:** Comparison of periods and light pulse responses in mice with different genotype

**Genotype**	**Periods**	**Light pulse ZT14**	**Light pulse ZT22**
		**[phase delay]**	**[phase advance]**
	**Hours (SEM)**	**Hours (SEM)**	**Hours (SEM)**
WT (n = 6)	23.6 (0.1)	1.3 (0.1)	0.8 (0.2)
PER1 (n = 4)	22.8 (0.2)	1.5 (0.2)	0.5 (0.2)
PER2 (n = 8)	arrhythmic	N.D.	N.D.
PML (n = 12)	23.2 (0.2)	1.0 (0.1)	0.9 (0.3)
PER1/PML (n = 23)	22.2 (0.2)	2.0 (0.2)	1.5 (0.3)
PER2/PML (n = 6)	arrhythmic	N.D.	N.D.

Next we examined whether the phase shift behavior of *Per1*^*−/−*^*/Pml*^*−/−*^ mice was different from that of wild type, *Pml*^*−/−*^ or *Per1*^*−/−*^ mice. A protocol in which the light pulse was given at either ZT14 or ZT22 on the last day of entrainment and prior to release into a free-running environment was used [8,9]. With this protocol, wildtype mice displayed a −1.3 ± 0.1 h and +0.8 h ± 0.2 h phase delay and phase advance response to ZT14 and ZT22 light pulses, respectively (Table 1). The *Per1*^*−/−*^ mice displayed a −1.5 ± 0.2 h and +0.5 h ± 0.3 h phase delay and phase advance response to ZT14 and ZT22 light pulses, respectively. The *Pml*^*−/−*^ mice displayed a −1.0 ± 0.1 h and +0.9 h ± 0.3 h phase delay and phase advance response to ZT14 and ZT22 light pulses, respectively. The *Per1*^*−/−*^*/Pml*^*−/−*^ mice displayed a −2.0 h ± 0.2 h and +1.5 h ± 0.3 h phase delay and phase advance response, respectively (Figure 2A-D, Table 1). These observations demonstrate that the *Per1*^*−/−*^*/Pml*^*−/−*^ mice have a significantly altered phase shift response, shorter average period length and increased advance of phase angle of entrainment when compared to wild type, *Pml*^*−/−*^ or *Per1*^*−/−*^ mice. Together, these behavioral observations indicate that the loss of PML and PER1 functions further altered the mammalian clock in vivo from that observed for the single gene mutant mice.

### Circadian behavior of *Per2*^*−/−*^*/Pml*^*−/−*^ mice

**Figure 4:**
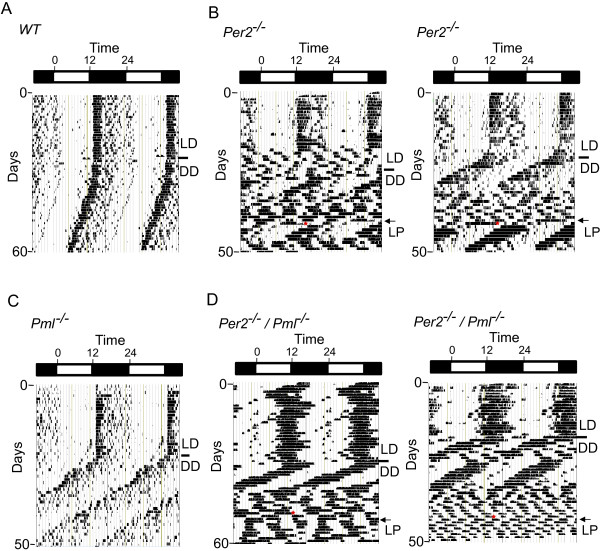
**Wheel-running behavior of *Per2***^***−/−***^**, *Pml***^***−/−***^**and *Per2***^***−/−***^***/Pml***^***−/−***^**mice.** The wheel-running actograms are double plotted showing a 12 h light/12 h dark cycle (LD) and a free-running period of 12 h dark/12 h dark (DD) cycles. The marker identifies the transition from LD to DD. Light pulse (LP) is indicated by the red asterisk. Actograms are representative of **(A)** a wild type mouse, **(B)** two representative *Per2*^*−/−*^ mice, **(C)** a representative *Pml*^*−/−*^ mouse, and **(D)** two representative *Per2*^*−/−*^**/***Pml*^*−/−*^ mice. Note the level of increased activity onset prior to ZT12 in the LD cycle.

Next we examined the circadian behavior of *Per2*^*−/−*^*/Pml*^*−/−*^ mice using a similar wheel-running analysis. When released into a free-running environment, some of the *Per2*^*−/−*^*/Pml*^*−/−*^ mice displayed a transient rhythm that deteriorated into arrhythmic activities (Figure 4A-C). These behaviors were also observed in *Per2*^*−/−*^ mice. Previous studies of *Per2*^*m/m*^ mice described a similar behavioral phenotype [6]. This similarity in the *Per2*^*−/−*^ and *Per2*^*−/−*^*/Pml*^*−/−*^ mice behavior suggests that the primary cause of the loss of rhythm behavioral phenotype in the free-running state is a loss of PER2 function. On the other hand, all arrhythmic *Per2*^*−/−*^ mice (n = 4) treated with a light pulse transiently returned to rhythmic behavior, as previously observed (Figure 4B) [9]. However, the transient post-light pulse rhythm seen in the arrhythmic *Per2*^*−/−*^ mice was not apparent in 50% of arrhythmic *Per2*^*−/−*^*/Pml*^*−/−*^ mice (n = 4) that were similarly treated (Figure 4C). These observations suggest that the endogenous clock of *Per2*^*−/−*^*/Pml*^*−/−*^ mice is further compromised from that of *Per2*^*−/−*^ mice.

The phase shift response of *Per2*^*−/−*^*/Pml*^*−/−*^ mice was also examined using a similar protocol in which a light pulse was given at either ZT14 or ZT22 on the last day of entrainment prior to release into a free-running environment. The phase shift response could not be determined accurately due to immediate loss of rhythm in many of the *Per2*^*−/−*^*/Pml*^*−/−*^ mice when released into a free-running condition. Comparing *Per2*^*−/−*^*/Pml*^*−/−*^ mice to *Per2*^*−/−*^ mice, it was apparent that the *Per2*^*−/−*^*/Pml*^*−/−*^ mice were much more active in the LD cycle (Figure 4C). The daily activity onset of *Per2*^*−/−*^*/Pml*^*−/−*^ mice in an LD cycle was highly variable and changed from day to day, unlike that seen in *Per2*^*−/−*^ mice (Figure 4B, C). On average, the phase angle of entrainment was about 2.5 h prior to ZT12 (Figure 3). This is significantly greater than the value of 0.4 h prior to ZT12 previously reported for *Per2*^*m/m*^ mice [9]. These observations suggest that the robustness of LD entrainment is affected and the phase angle of entrainment is also advanced when mice are deficient in both PER2 and PML when compared to PER2 or PML deficient mice.

## Discussion

Core clock genes are primarily defined by the altered circadian behavioral phenotype when the gene is mutated in an organism. In the current paradigm, a core clock gene deficiency is likely to disrupt circadian behavior in a drastic manner, while a clock-controlled gene deficiency is unlikely to have an impact on circadian behavior. However, in mammals, this differentiation is complicated by the presence of redundant homologs of core clock genes. Existing evidence appears to show that single gene mutations in mice rarely result in a total disruption of the endogenous clock. One possible exception is *Bmal1* and to a lesser extent *Per2*, where a loss of function mutation can result in arrhythmic phenotypes [6,10]. Surprisingly, constitutive expression of *Bmal2* in *Bmal1*^*−/−*^ mice restored circadian rhythmicity [11]. The loss of rhythm phenotype of *Bmal1*^*−/−*^ is linked to BMAL1 regulation of *Bmal2* expression. For other identified core clock genes in mice, gene redundancy prevents loss of circadian rhythm when a key clock gene is mutated. In some instances, the endogenous clock rhythm can be as robust as wildtype, such as in CLOCK or NPAS2 null deficiency [12,13]. Similar findings were also observed for REV-ERBα or REV-ERBβ deficiency [14]. Other outcomes seen in core clock gene deficiency are shortening or lengthening of period length, as in the loss of CRY1 and CRY2, respectively. The characteristics of PER1 and PER2 deficiency phenotype is an unstable period and a clock with a transient shortened period that lose persistence in rhythm, respectively. In free-running conditions, double knockout mice with either CLOCK/NPAS2 [15], REV-ERBα/REV-ERBβ [14], CRY1/CRY2 [16] or PER1/PER2 [4] deficiency displayed arrhythmic locomotor behavior indicating that the endogenous clock machinery was completely disrupted. The loss of DEC1 (SHARP2) and its redundant paralog DEC2 (SHARP1) mildly impacted clock entrainment [17]. At a molecular level, decreased expression amplitude of key clock regulators in the SCN and peripheral organs was observed. The phenotype and molecular profiles of DEC1 (SHARP2) and DEC2 (SHARP1) deficient mice are consistent with a central clock mechanism that is largely intact. Possible explanations are that these are players in a minor clock regulatory loop or perhaps they are clock-controlled genes.

The clock behavioral rhythm based on wheel-running activities revealed that mice deficient in both PML and PER1 have significant abnormalities of their endogenous clock. Some of the circadian behavior abnormality appeared significantly different from circadian behaviors of either *Pml*^*−/−*^, *Per1*^*−/−*^ or wild type mice. Two previous studies showed that the phase delay of our *Per1*^*−/−*^ mice was similar to that of wildtype mice, while the phase advance was moderately reduced [8,9]. Our new analysis confirmed these previous observations. In contrast, the *Pml*^*−/−*^/*Per1*^−/−^ mice described here displayed significant differences in period length, phase shift response and phase angle of entrainment from those of *Pml*^*−/−*^, *Per1*^*−/−*^ or wild type mice.

The arrhythmic free-running behaviors of *Per2*^*−/−*^*/Pml*^*−/−*^ mice are similar to those of *Per2* deficient mice [6]. However, the restoration of transient rhythm by a light pulse during arrhythmic behavior of *Per2*^*−/−*^ mice in a free-running state was less apparent in *Per2*^*−/−*^*/Pml*^*−/−*^ mice [9]. In addition, *Per2*^*−/−*^*/Pml*^*−/−*^ mice displayed a significantly more advanced phase angle of entrainment in an LD cycle than *Per2*^*−/−*^ mice. Together, the behavioral analysis suggests that the loss of PML caused additional perturbation to the already compromised *Per2*^*−/−*^ or *Per1*^*−/−*^ clock mechanism.

Our previous studies showed that loss of PML significantly decreased PER2 nuclear entry, which in turn reduced its interaction with BMAL1 and CLOCK [3]. Other studies have indicated that PML directly interacts with BMAL1 [18]. These observations suggest that PML likely modulates BMAL1/CLOCK mediated transcription of its target genes. Indeed, using *Per1*-Luc reporter assay, we observed that PML enhanced BMAL1/CLOCK mediated transcription of the *Per1* promoter (data not shown). In addition to BMAL1 and PER2, a growing list of PML interacting proteins including SIRT1, CBP, WDR5 and SIN3A have been reported to interact with key clock regulators or be involved in the circadian clock mechanism [19-22]. Thus, the additional clock behavioral defects of *Per2*^*−/−*^*/Pml*^*−/−*^ or *Per1*^*−/−*^*/Pml*^*−/−*^ mice, in contrast to the individual gene deficient mice, suggest that PML likely regulates additional regulators of the mammalian clock mechanism.

## Conclusions

The current studies show that the circadian behavior of *Per1*^*−/−*^*/Pml*^*−/−*^ and *Per2*^*−/−*^*/Pml*^*−/−*^ mice differs significantly from that of *Per1*^*−/−*^*, Per2*^*−/−*^ and *Pml*^*−/−*^ mice, suggesting that PML has additional roles in mouse circadian clock beyond merely regulating PER1 and PER2. These observations based on behavioral analysis suggest that PML’s regulatory role in the mouse clock mechanism may involve multiple targets.

## Competing interest

These studies were undertaken with NIH funding to CCL. All authors were employed by University of Texas at the time when these studies were carried out. There is not competing interest to declare.

## Authors’ contributions

TM conducted the majority of the experiments and data analysis, and also contributed to manuscript preparation; MC-Goodspeed contributed to the generation of *Per2*^*−/−*^*/Pml*^*−/−*^ mice and their initial characterization. ZZ generated *Per1*^*−/−*^*/Pml*^*−/−*^ mice, performed wheel-running experiments with *Per1*^*−/−*^ mice, and contributed to the preparation of the manuscript. CCL directed the study, analyzed the data and prepared the manuscript. All authors read and approved the final manuscript.

## References

[B1] Salomoni P Pandolfi PP The role of PML in tumor suppression Cell 2002 108 165 170 10.1016/S0092-8674(02)00626-8 11832207

[B2] Bernardi R Pandolfi PP Structure, dynamics and functions of promyelocytic leukaemia nuclear bodies Nat Rev Mol Cell Biol 2007 8 December 1006 1016 17928811 10.1038/nrm2277

[B3] Miki T Xu Z Chen-Goodspeed M Liu M Van Oort-Jansen A Rea M a Zhao Z Lee CC Chang K-S PML regulates PER2 nuclear localization and circadian function EMBO J 2012 31 1427 1439 10.1038/emboj.2012.1 22274616 PMC3321181

[B4] Zheng B Albrecht U Kaasik K Sage M Lu W Vaishnav S Li Q Sun ZS Eichele G Bradley A Lee CC Nonredundant roles of the mPer1 and mPer2 genes in the mammalian circadian clock Cell 2001 105 683 694 10.1016/S0092-8674(01)00380-4 11389837

[B5] Cermakian N Monaco L Pando MP Dierich A Sassone-corsi P Altered behavioral rhythms and clock gene expression in mice with a targeted mutation in the Period1 gene EMBO J 2001 20 3967 3974 10.1093/emboj/20.15.3967 11483500 PMC149149

[B6] Zheng B Larkin DW Albrecht U Sun ZS Sage M Eichele G Lee CC Bradley A The mPer2 gene encodes a functional component of the mammalian circadian clock Nature 1999 400 169 173 10.1038/22118 10408444

[B7] Gang Wang Z Role of PML in cell growth and the retinoic acid pathway Science 1998 279 1547 1551 10.1126/science.279.5356.1547 9488655

[B8] Albrecht U Zheng B Larkin D Sun ZS Lee CC mPer1 and mPer2 are essential for normal resetting of the circadian clock J Biol Rhythms 2001 16 100 104 10.1177/074873001129001791 11302552

[B9] Spoelstra K Albrecht U Van der Horst GTJ Brauer V Daan S Phase responses to light pulses in mice lacking functional per or cry genes J Biol Rhythms 2004 19 518 529 10.1177/0748730404268122 15523113

[B10] Bunger MK Wilsbacher LD Moran SM Clendenin C Radcliffe LA Hogenesch JB Simon MC Takahashi JS Bradfield CA Mop3 is an essential component of the master circadian pacemaker in mammals Cell 2000 103 1009 1017 10.1016/S0092-8674(00)00205-1 11163178 PMC3779439

[B11] Shi S Hida A Mcguinness OP Wasserman DH Yamazaki S Circadian clock gene Bmal1 is not essential; functional replacement with its Paralog, Bmal2 Curr Biol 2010 20 316 321 10.1016/j.cub.2009.12.034 20153195 PMC2907674

[B12] Debruyne JP Noton E Lambert CM Maywood ES Weaver DR Reppert SM A clock shock: mouse CLOCK is not required for circadian oscillator function Neuron 2006 50 465 477 10.1016/j.neuron.2006.03.041 16675400

[B13] Garcia J a Impaired cued and contextual memory in NPAS2-deficient Mice Science 2000 288 2226 2230 10.1126/science.288.5474.2226 10864874

[B14] Cho H Zhao X Hatori M Yu RT Barish GD Lam MT Chong L Ditacchio L Atkins AR Glass CK Liddle C Auwerx J Downes M Panda S Evans RM Regulation of circadian behaviour and metabolism by REV-ERB-α and REV-ERB-β Nature 2012 485 123 127 10.1038/nature11048 22460952 PMC3367514

[B15] DeBruyne JP Weaver DR Reppert SM CLOCK and NPAS2 have overlapping roles in the suprachiasmatic circadian clock Nat Neurosci 2007 10 543 545 10.1038/nn1884 17417633 PMC2782643

[B16] Vitaterna MH Selby CP Todo T Niwa H Thompson C Fruechte EM Hitomi K Thresher RJ Ishikawa T Miyazaki J Takahashi JS Sancar A Differential regulation of mammalian period genes and circadian rhythmicity by cryptochromes 1 and 2 Proc Natl Acad Sci 1999 96 12114 12119 10.1073/pnas.96.21.12114 10518585 PMC18421

[B17] Rossner MJ Oster H Wichert SP Reinecke L Wehr MC Reinecke J Eichele G Taneja R Nave K Disturbed clockwork resetting in Sharp-1 and Sharp-2 single and double mutant mice PLoS One 2008 3 e2762 10.1371/journal.pone.0002762 18648504 PMC2447179

[B18] Lee J Lee Y Lee MJ Park E Kang SH Chung CH Lee KH Kim K Dual modification of BMAL1 by SUMO2/3 and ubiquitin promotes circadian activation of the CLOCK/BMAL1 complex Mol Cell Biol 2008 28 6056 6065 10.1128/MCB.00583-08 18644859 PMC2546997

[B19] Brown SA Ripperger J Kadener S Fleury-Olela F Vilbois F Rosbash M Schibler U PERIOD1-associated proteins modulate the negative limb of the mammalian circadian oscillator Science 2005 308 6 10.1126/science.1107373 15860628

[B20] Nakahata Y Kaluzova M Grimaldi B Sahar S Hirayama J Chen D Guarente LP Sassone-Corsi P The NAD + −dependent deacetylase SIRT1 modulates CLOCK-mediated chromatin remodeling and circadian control Cell 2008 134 329 340 10.1016/j.cell.2008.07.002 18662547 PMC3526943

[B21] Lee Y Lee J Kwon I Nakajima Y Ohmiya Y Son GH Lee KH Kim K Coactivation of the CLOCK-BMAL1 complex by CBP mediates resetting of the circadian clock J Cell Sci 2010 123 Pt 20 3547 3557 20930143 10.1242/jcs.070300

[B22] Duong HA Robles MS Knutti D Weitz CJ A molecular mechanism for circadian clock negative feedback Science 2011 332 1436 1439 10.1126/science.1196766 21680841 PMC3859310

